# PLK1 protects intestinal barrier function during sepsis by targeting mitochondrial dynamics through TANK-NF-κB signalling

**DOI:** 10.1186/s10020-022-00597-z

**Published:** 2022-12-29

**Authors:** Ying-Ya Cao, Yuan Zhang, Wuyun Gerile, Yan Guo, Li-Na Wu, Li-Li Wu, Kai Song, Wei-Hua Lu, Jian-Bo Yu

**Affiliations:** 1 Department of Anaesthesiology and Critical Care Medicine, Tianjin Nankai Hospital, Tianjin Medical University, Tianjin, 300100 China; 2 Department of Critical Care Medicine, The First Affiliated Hospital of Wannan Medical College, Wuhu, 241001 Anhui China

**Keywords:** Sepsis, Intestinal barrier, Apoptosis, Mitochondrial dynamics, PLK1, TANK, Nuclear factor-κB

## Abstract

**Background:**

Intestinal barrier integrity in the pathogenesis of sepsis is critical. Despite an abundance of evidence, the molecular mechanism of the intestinal barrier in sepsis pathology remains unclear. Here, we report a protective role of polo-like kinase 1 (PLK1) in intestinal barrier integrity during sepsis.

**Methods:**

Mice with PLK1 overexpression (CAG-PLK1 mice) or PLK1 inhibition (BI2536-treated mice) underwent caecal ligation and puncture (CLP) to establish a sepsis model. The intestinal barrier function, apoptosis in the intestinal epithelium, mitochondrial function and NF-κB signalling activity were evaluated. To suppress the activation of NF-κB signalling, the NF-κB inhibitor PDTC, was administered. The Caco-2 cell line was chosen to establish an intestinal epithelial injury model in vitro.

**Results:**

Sepsis destroyed intestinal barrier function, induced excessive apoptosis in the intestinal epithelium, and disrupted the balance of mitochondrial dynamics in wild-type mice. PLK1 overexpression alleviated sepsis-induced damage to the intestinal epithelium by inhibiting the activation of NF-κB signalling. PLK1 colocalized and interacted with TANK in Caco-2 cells. Transfecting Caco-2 cells with TANK-SiRNA suppressed NF-κB signalling and ameliorated mitochondrial dysfunction, apoptosis and the high permeability of cells induced by lipopolysaccharide (LPS). Furthermore, TANK overexpression impaired the protective effect of PLK1 on LPS-induced injuries in Caco-2 cells.

**Conclusion:**

Our findings reveal that the PLK1/TANK/NF-κB axis plays a crucial role in sepsis-induced intestinal barrier dysfunction by regulating mitochondrial dynamics and apoptosis in the intestinal epithelium and might be a potential therapeutic target in the clinic.

**Supplementary Information:**

The online version contains supplementary material available at 10.1186/s10020-022-00597-z.

## Introduction

Sepsis develops due to a dysregulated host response to infection and can lead to life-threatening organ dysfunction (Evans et al. 2021). Of note, this syndrome is a challenge for public health for its increasing morbidity and approximately 30% mortality (Vincent et al. 2019; Bauer et al. 2020). The intestine is vulnerable during sepsis presented as disrupted mucus integrity and increased mucosal permeability, thus allows the transport of toxic mediators to the blood circulation and leads to distant organ dysfunction (Mittal et al. 2014; Klingensmith et al. 2016).

The intestinal mucosa consists of a single-layered epithelium that is highly dynamic and precisely regulated (Peterson et al. 2014; Odenwald et al. 2017). The balance between proliferation and apoptosis in the epithelium maintains intestinal homeostasis. Excessive apoptosis in the intestinal epithelium can damage epithelial integrity, resulting in intestinal hyperpermeability (Yoseph et al. 2016). As energy-generating organelles, mitochondria are also involved in regulating apoptosis, calcium homeostasis, and oxidative metabolism (Nunnari et al. 2012). Mitochondria are dynamic organelles that undergo fusion and fission, which not only affects their morphology but is also linked with their function. During sepsis, mitochondria undergo excessive fission and insufficient fusion, which damages mitochondrial quality and function, causing cellular dysfunction (Parikh et al. 2015; Chan 2020). Treating the imbalance in mitochondrial fusion and fission will improve therapeutic options for sepsis.

Sepsis is a series of systemic inflammatory responses accompanied by the activation of nuclear factor-κB (NF-κB) signalling. The role of NF-κB signalling in sepsis is controversial for its activation contributes to inflammatory injury in tissues whereas it also mediates host defence and other protective cellular responses (Stone et al. 2017; Ren et al. 2020; Yang et al. 2020; Xiao et al. 2021). Because NF-κB acts as an oxidative stress response transcription factor, it is not surprising that NF-κB signalling affects mitochondrial morphology and function (Laforge et al. 2016; Huang et al. 2020). However, the specific role of NF-κB signalling in mitochondrial fusion and fission in the intestinal epithelium during sepsis deserves further examination.

Polo-like kinase 1 (PLK1), a well-known regulator of mitosis, participates in the G2/M transition, DNA integrity maintenance, and the maturation of various organelles (Gutteridge et al. 2016; Ciardo et al. 2020; Shakeel et al.^2021^). Our previous studies revealed that PLK1 overexpression could rescue lipopolysaccharide (LPS)-induced apoptosis in different types of cells in vitro (Cao et al. 2018, 2021), but little is known about its role during sepsis in vivo. Furthermore, PLK1 has been reported to reduce the nuclear translocation of NF-κB in vitro, but the underlying mechanism is unknown (Cao et al. 2020). TRAF family member-associated NF-κB activator (TANK) is a TRAF-binding protein that is an upstream regulator of NF-κB signalling. An earlier study revealed the interaction of PLK1 with TANK in HEK293 cells (Zhang et al. 2010). Thus, we hypothesize that PLK1 regulates NF-κB activity by interacting with TANK in the intestinal epithelium.

Here, we examined the role of PLK1 in sepsis-induced intestinal injury by overexpressing or inhibiting PLK1 in vivo. Furthermore, we explored the potential mechanism of PLK1 on sepsis-induced intestinal injury in vivo and in vitro. These findings clarify the role of PLK1/TANK/NF-κB axis and suggest a novel therapeutic target for the treatment of sepsis-induced intestinal barrier dysfunction.

## Materials and methods

### Reagents and antibodies

Lipopolysaccharide (LPS, L2880) was purchased from Sigma–Aldrich (St. Louis, MO, USA), BI2536 (S1109) and pyrrolidinedithiocarbamate ammonium (PDTC, S3633) were purchased from Selleck Chemicals (Houston, TX, USA). Goat anti-mouse (31,430,100) and goat anti-rabbit (31,460,100) secondary antibodies were purchased from Invitrogen (Carlsbad, CA, USA). The primary antibodies used in the study are listed in Additional file 1: Table S1.

### Animal preparation and sepsis model

All experimental protocols were performed according to the Guide for Laboratory Animal Care and Use and were approved by the Institutional Animal Care and Ethics Committee of Wannan Medical College (LLSC-2020–074).

Wild-type (WT) male C57BL/6J mice (aged 6–8 weeks, weighing 20–25 g) were purchased from GemPharmatech Co., Ltd. (Nanjing, China). Rosa26-CAG-CreERT2;H11-Loxp-Stop-Loxp-PLK1 mice (PLK1 conditional knock-in mice, hereafter referred to as CAG-PLK1 mice) and CAG-CreERT2 mice were generated by Shanghai Model Organisms Centre, Inc. All mice were maintained with a 12 h light/dark cycle in a specific pathogen-free (SPF) animal room with access to water and a standard rodent diet. Tamoxifen (Sigma–Aldrich) was dissolved in corn oil at a concentration of 20 mg/ml, and intraperitoneal injection of 100 µl was used once per day for a total of five consecutive days to induce conditional knock-in PLK1 in mice. The experiments were started 1 week after the final injection.

Caecal ligation and puncture (CLP) was performed to establish the septic mouse model as previously described (Rittirsch et al. 2009). Briefly, the mice were anaesthetised with 2% isoflurane, and a 0.5–1 cm midline abdominal incision was performed to expose the caecum. Subsequently, the caecum was ligated below the ileocecal valve in the distal three-quarters of the caecum, followed by a single ‘through and through’ perforation with a 21-gauge needle. After being punctured, the caecum was returned to the abdomen, and the incision was sutured with sterile 3–0 silk. Sham-operated mice were subjected to laparotomy without CLP. Saline solution was injected subcutaneously (50 mg/kg) for resuscitation after the surgery. The mice were then randomly assigned to different groups (n = 6/per group) according to various objectives, and the details are presented in Table 1.

**Table 1:** Experimental treatments and groupings of the mice

Group	Treatment
Sham	WT mice with sham operation
CLP	WT mice with CLP operation
PLK1-Sham	CAG-PLK1 mice with sham operation
PLK1-CLP	CAG-PLK1 mice with CLP operation
BI-Sham	WT mice that were intraperitoneal injected with BI2536 (10 mg/kg BW) 24 h before sham operation
BI-CLP	WT mice that were intraperitoneal injected with BI2536 (10 mg/kg BW) 24 h before CLP operation
PDTC-Sham	WT mice that were intraperitoneal injected with PDTC (100 mg/kg BW) 2 h before sham operation
PDTC-CLP	WT mice that were intraperitoneal injected with PDTC (100 mg/kg BW) 2 h before CLP operation

### Enzyme-linked immunosorbent assay (ELISA)

At 24 h after the surgery, the mice were anaesthetized, and blood was collected from the heart. Then, the mice were euthanized, and the blood was centrifuged at 3000×*g* for 15 min at 4 °C. The serum was collected, and the indicated parameters were analysed by ELISA kits (Cloud-clone, Wuhan, China) according to the manufacturer’s instructions.

### Haematoxylin and eosin (H&E) staining

Small intestine samples were collected 24 h after CLP and fixed in 10% paraformaldehyde overnight. The samples were then embedded in paraffin and sliced into 5 μm-thick sections. For pathological examinations, the sections were stained with H&E as previously described (Cao et al. 2018). The severity of mucosal injury was assessed with Chiu’s scoring system as previously described (Chiu et al. 1970).

### Terminal deoxynucleotidyl transferase (TdT)-mediated dUDP-biotin nick end labelling (TUNEL) staining

To examine apoptotic cells in the intestinal epithelium, a TUNEL assay was performed as previously described (Cao et al. 2018). The tissue sections were incubated in a permeabilization solution, stained with the reaction mixture and subsequently incubated with DAPI. TUNEL-positive cells were characterized by dark brown staining of the nucleus and nuclear membrane.

### Cell culture and treatments

The human intestinal Caco-2 cell line was purchased from Cellcook Biotech (Guangzhou, China) and cultured in DMEM supplemented with 10% foetal bovine serum (FBS) (Invitrogen, San Diego, CA, USA), 100 U/ml penicillin and 100 mg/ml streptomycin at 37 °C in a humidified atmosphere of 5% CO_2_. When the cells reached 70–80% confluence, the cells were exposed to various treatments according to various objectives.

### Transfection with plasmids and siRNA

The plasmids pCDNA3.1-PLK1, pCDNA3.1-TANK and Si-TANK (5’- GAACUAUGAGCAGAGAAUATT-3’) were constructed by Keygen Biotech (Nanjing, China), along with the corresponding controls. Caco-2 cells were cultured in 6-well plates, and when they reached 60–70% confluence, the original media was replaced with serum-free Opti-MEM. Subsequently, the plasmids or siRNA was transfected into the cells using Lipofectamine 3000 (Invitrogen, Carlsbad, CA, USA) following the instructions.

### Apoptosis analysis

Apoptotic cells were double-labelled with Annexin V–APC and 7-AAD using an Annexin V-APC/7-AAD kit (KGA1024, Keygen Biotech, China) and analysed with a BDTM LSRΙΙ flow cytometer (BD Biosciences). Annexin V-positive cells were counted and defined as apoptotic cells.

### Measurement of reactive oxygen species (ROS) production

To measure ROS generation, tissue slides or cells were incubated with 10 µM DCFH-DA (KGT010-1, Keygen Biotech, China), which is a fluorescent ROS probe at 37 °C for 20 min. Then, the cells were washed with phosphate-buffered saline (PBS) 3 times, and the nuclei were stained with Hoechst. All procedures were performed in the dark, and the samples were observed by a laser scanning confocal microscope (Olympus, Tokyo, Japan).

### Measurement of mitochondrial membrane potential (MMP)

MMP was measured using a JC-1 MMP assay kit (KGA602, Keygen Biotech, China). Fluorescence images were acquired using a fluorescence microscope (Olympus, Tokyo, Japan) with an excitation wavelength of 488 nm and emission wavelengths of 529 and 590 nm for JC-1 monomers and aggregates, respectively. The loss of MMP was reflected by the ratio of aggregates (red fluorescence) to monomers (green fluorescence).

### Measurement of transepithelial electrical resistance (TEER)

TEER was measured as described previously (Zheng et al. 2020). Briefly, Caco-2 cells were seeded on the upper layer of a Transwell 6-well plate (0.4 μm, Corning, America), and different treatments were added according to the experimental design. Then, an epithelial volt–ohm metre with a chopstick electrode (World Precision Inc, America) was used to measure the TEER every 1 h.

### Immunoprecipitation assay

To examine the endogenous interaction between PLK1 and TANK, Caco-2 cells were washed with ice-cold PBS and lysed in IP lysis buffer (Beyotime, China) supplemented with PMSF (Beyotime, China). The lysates were then centrifuged and incubated with Protein A/G agarose beads (Santa Cruz, CA, USA), after which the beads were cleared by centrifugation. Lysates and immunoprecipitates were incubated with the indicated primary antibodies and the appropriate secondary antibodies, followed by separation by SDS–PAGE and detection.

### Transmission electron microscopy (TEM)

TEM was used to evaluate the ultrastructure of the intestine and cells according to a previous study (Shi et al. 2021). The small intestine or cells were fixed in 2.5% glutaraldehyde for 3 h and treated with 1% osmium tetroxide for 1 h. After being dehydrated in a graded ethanol series, the tissues were embedded in epoxy resin, sliced with an ultramicrotome, and subsequently stained with uranyl acetate and lead citrate. Finally, the samples were observed and analysed under a transmission electron microscope.

### Immunofluorescence staining

For immunofluorescence staining, intestine tissue slices and cells were fixed with 4% paraformaldehyde for 10 min, washed with PBS, incubated with primary antibodies overnight at 4 °C, incubated with secondary antibodies at room temperature in the dark, washed with PBS three times and imaged using a fluorescence microscope (Olympus, Tokyo, Japan).

### Cell lysis and protein extraction

The small intestinal epithelium was isolated as previously described (Peuker et al. 2016), and then total proteins were extracted with a protein extraction kit (Beyotime, China). To extract total proteins from the cells, the harvested cells were washed with cold PBS and lysed with lysis buffer containing protease and phosphatase inhibitor cocktails. A nuclear extraction kit (Sigma–Aldrich, MO, USA) was used for nuclear-cytoplasmic fractionation. The protein concentration was quantified with a BCA protein assay kit (Beyotime, China).

### Immunoblotting

To determine the levels of the indicated proteins, equal amounts of protein were separated by SDS–PAGE and transferred to PVDF membranes (Millipore, Bedford, MA, USA). Subsequently, the membranes were blocked with 5% milk in Tris-buffered saline-Tween 20 at room temperature for 1 h and then probed with the indicated primary antibodies overnight at 4 °C. After being washed with PBS three times, the membranes were incubated with the corresponding secondary antibodies. Finally, the membranes were analysed using the super ECL detection reagent (Tanon, Guangzhou, China).

### Statistical analysis

Prism 5.0 (GraphPad Software Inc, San Diego, CA, USA) was used for data analysis. Quantitative data are presented as the mean ± SD, and the data were statistically evaluated using Student’s t tests or one-way ANOVA. A value of p < 0.05 was considered statistically significant.

## Results

### Sepsis induces intestinal injury and reduces PLK1 expression in the intestinal epithelium

**Fig. 1:**
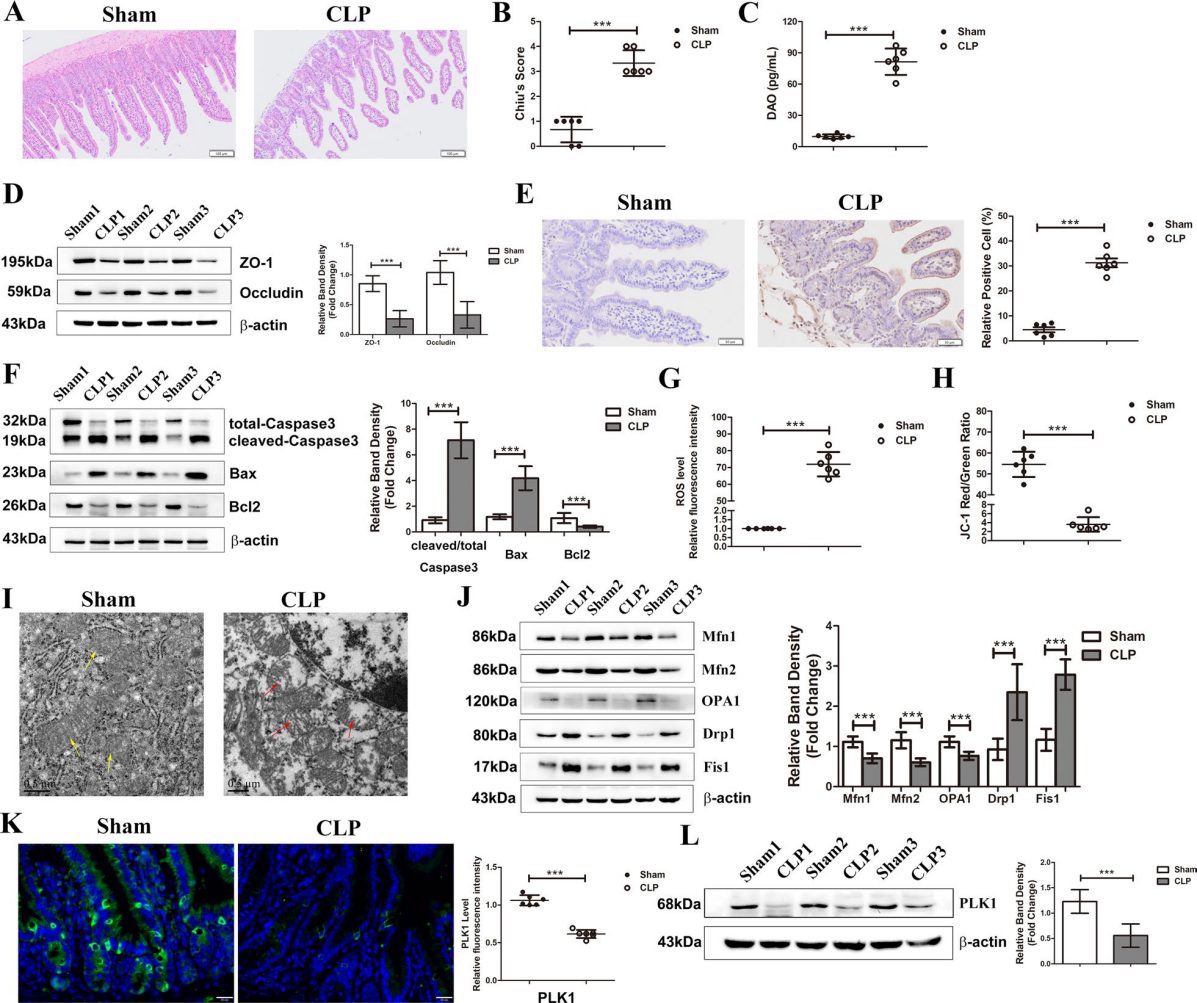
Sepsis induces intestinal injury and reduces PLK1 expression in the intestinal epithelium. The mice were sacrificed 24 h after CLP and then analysed. **A** Microphotographs of hematoxylin and eosin (H&E) stained sections of intestine. Scale bar indicates 100 μm. **B** The Chiu’s scores of each group. **C** The serum concentration of DAO for each group. **D** The levels of ZO-1 and Occludin were analyzed with Western blot. The graph shows the relative band densities. **E** Representative images of TUNEL staining. The right graph represented the percentage of positive TUNEL stained cell. Scale bar indicates 50 μm. **F** The levels of apoptotic markers in intestine 24 h after CLP were measured. The right graph shows the relative band densities. **G** The relative fluorescence intensity of ROS in the intestine. **H** The ratio of JC-1 red/green fluorescence intensity which reflecting the MMP level was shown in graph. **I** Morphological alterations in mitochondria were examined by TEM (scale bar = 0.5 μm). Yellow arrows indicate the relatively normal mitochondrial shape in the sham group. Red arrows denote deformed mitochondria with the loss of clearly defined cristae. **J** Representative Western blots showing mitochondrial fusion (Mfn1, Mfn2, OPA1) and fission (Drp1 and Fis1) markers on the left, and the graph on the right shows the relative band densities. **K** Representative PLK1 immunohistochemical staining in the intestine (scale bar = 20 μm). The graph shows the relative fluorescence intensity. **L** Representative Western blots presents the expression of PLK1 in intestine and the graph on the right shows the relative band densities. The data in the graphs are expressed as the mean ± SD. ***P < 0.001 versus the control group (n = 6)

To confirm the effect of CLP on intestinal barrier function in mice, intestine slides were stained with H&E and evaluated by Chiu’s scoring. The intestinal villi in CLP mice were atrophic, and the scores were increased (Fig. 1A, B). Furthermore, the serum levels of diamine oxidase (DAO), which reflects intestinal permeability, were increased in CLP mice (Fig. 1C). The expression levels of ZO-1 and Occludin, which are two tight junction proteins in the intestinal epithelium, were markedly decreased in CLP mice (Fig. 1D). The function of the intestinal barrier largely depends on the balance in proliferation and apoptosis in the intestinal epithelium. Next, apoptosis in the intestinal epithelium was measured by TUNEL staining. CLP mice had elevated numbers of positively stained cells (Fig. 1E), along with increased expression of the proapoptotic proteins cleaved-Caspase3 and Bax and decreased level of antiapoptotic protein Bcl2 (Fig. 1F).As mitochondria coordinate cellular homoeostasis for their various roles in energy production, oxidative metabolism and apoptosis induction, mitochondrial function plays a critical role in intestinal barrier integrity (Rath et al. 2018). In this study, the production of ROS increased with decreasing MMP in CLP mice (Fig. 1G, H). The mitochondrial structure observed by electron microscopy shown that the mitochondrial ultrastructure was deformed and intact cristae were lost in the intestinal tissue of CLP mice (Fig. 1I). Moreover, Mitochondria are dynamic organelles, and the balance between mitochondrial fusion and fission is crucial for their function. We then measured the expression of related mitochondrial fusion/fission proteins in intestinal tissues. Compared with those in the sham group, the levels of mitochondrial fusion proteins (Mfn1, Mfn2, OPA1) were reduced, while the levels of mitochondrial fission proteins (Drp1, Fis1) were enhanced in mice with CLP (Fig. 1J), indicating a shift towards fission. Though our previous study found the decreased expression of PLK1 in sepsis (Cao et al. 2018, 2021), we also examined the level of PLK1 in intestinal epithelium during sepsis. Immunofluorescence staining and immunoblotting both showed lower expression of PLK1 in intestinal tissues in CLP mice (Fig. 1K, L).

### PLK1 overexpression ameliorates sepsis-induced intestinal injury

**Fig. 2:**
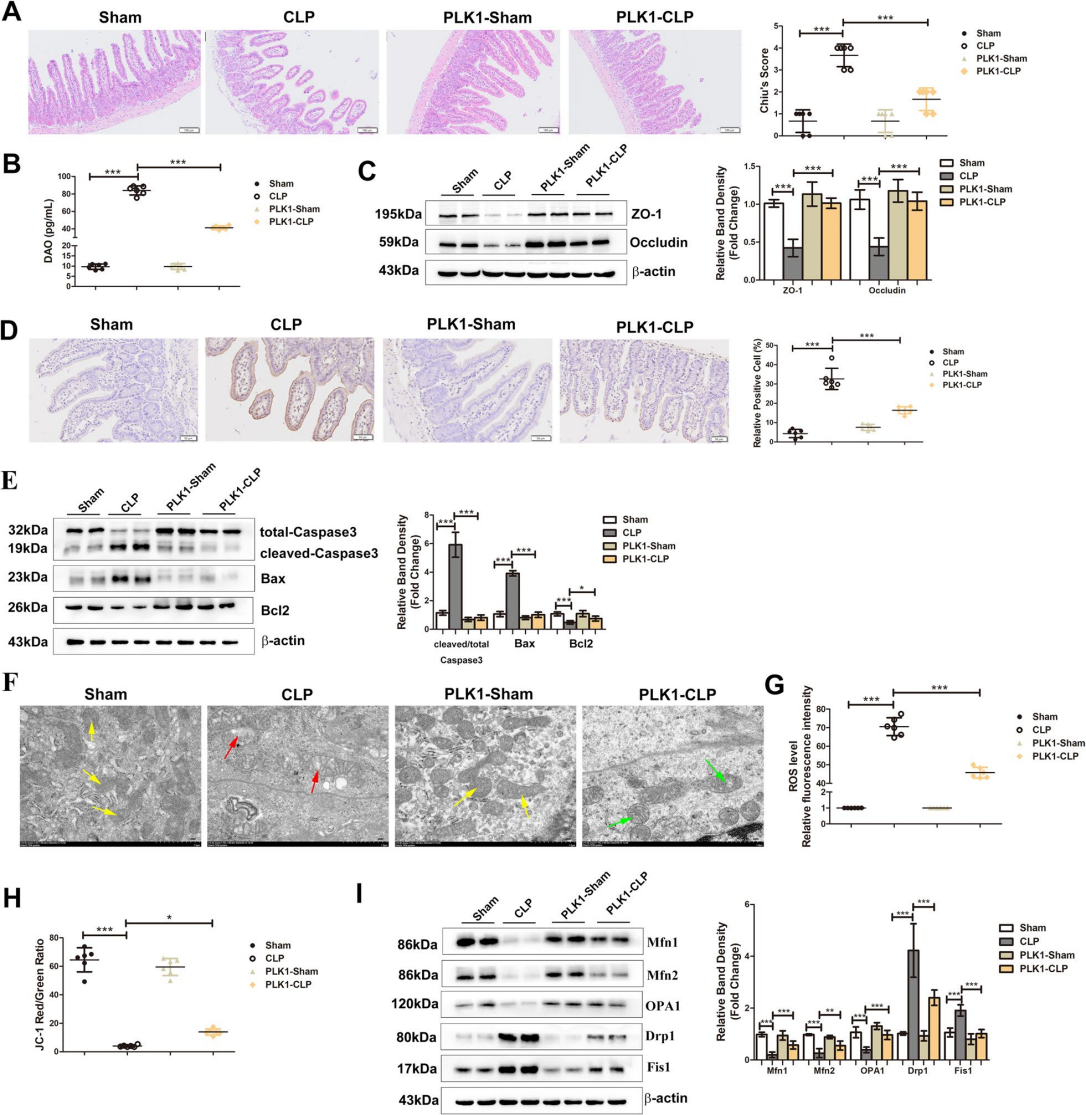
PLK1 overexpression ameliorates sepsis-induced intestinal injury. CAG-PLK1 mice or wild type mice were underwent CLP and then analysed. **A** Microphotographs of H&E-stained intestine sections from the indicated groups. The scale bar represents 100 μm. The Chiu’s score of each group shown right. **B** Serum concentrations of DAO in each group. **C** The levels of ZO-1 and Occludin were analysed by Western blotting. The graph shows the relative band densities. **D** Representative images of TUNEL staining after the indicated treatments. The graph shows the percentage of TUNEL-positive cells. The scale bar represents 50 μm. **E** The levels of apoptotic markers were measured by Western blots. The graph shows the relative band densities. **F** Morphological alterations in mitochondria were examined by TEM (scale bar = 1.0 μm). Yellow arrows indicate the relatively normal mitochondrial shape in the sham group. Red arrows denote deformed mitochondria with the loss of clearly defined cristae. Green arrows indicate rescued mitochondrial shape. **G** The relative fluorescence intensity of ROS in the intestine. **H** The ratio of JC-1 red/green fluorescence intensity which reflecting the MMP level was shown in graph. **I** Representative Western blots showing mitochondrial fusion (Mfn1, Mfn2, OPA1) and fission (Drp1 and Fis1) on the left, and the right graph show the relative band densities. The data in the graphs are expressed as the mean ± SD. *P < 0.05, **P < 0.01, ***P < 0.001 (n = 6)

To further explore the role of PLK1 in sepsis-induced intestinal barrier dysfunction, CAG-PLK1 mice were constructed and subjected to CLP. Compared with that in the control groups, the integrity of the intestinal barrier in septic CAG-PLK1 mice was ameliorated, and these mice had lower Chiu’s scores (Fig. 2A), decreased serum DAO levels (Fig. 2B) and increased expression of tight junction proteins (ZO-1 and Occludin) (Fig. 2C). Furthermore, the role of PLK1 in enterocyte apoptosis and mitochondrial function was also examined. Compared with that of control mice, the intestinal epithelia of septic CAG-PLK1 mice exhibited fewer TUNEL-positive cells, lower expression of cleaved-Caspase3 and Bax, and higher level of Bcl2 (Fig. 2D, E). Moreover, the damage to mitochondrial ultrastructure was ameliorated (Fig. 2F), ROS production was decreased (Fig. 2G), the MMP increased (Fig. 2H), the expression of mitochondrial fusion proteins (Mfn1, Mfn2, OPA1) increased, and the levels of mitochondrial fission proteins (Drp1, Fis1) decreased (Fig. 2I).To further verify the protective effect of PLK1, the selective PLK1 inhibitor BI2536 was intraperitoneally injected 24 h before CLP, and intestinal barrier function was assessed as previously described. The effects of the different treatments on PLK1 expression were also examined (Additional file 1: Fig. S1A, B). The results showed that the integrity of the intestinal barrier was worsened by PLK1 inhibition, as indicated by increased Chiu scores (Additional file 1: Fig. S1C), enhanced serum DAO levels (Additional file 1: Fig. S1D) and decreased expression of tight junction proteins (ZO-1 and Occludin) (Additional file 1: Fig. S1E). Moreover, the apoptosis in the intestinal epithelium (Additional file 1: Fig. S1F, G) and the mitochondrial function (Additional file 1: Fig. S1H–K) worsened in BI2536-treated CLP mice. These results indicate the beneficial effect of PLK1 on sepsis-induced intestinal injury.

### NF-κB signalling activation induces intestinal injury during sepsis

**Fig. 3:**
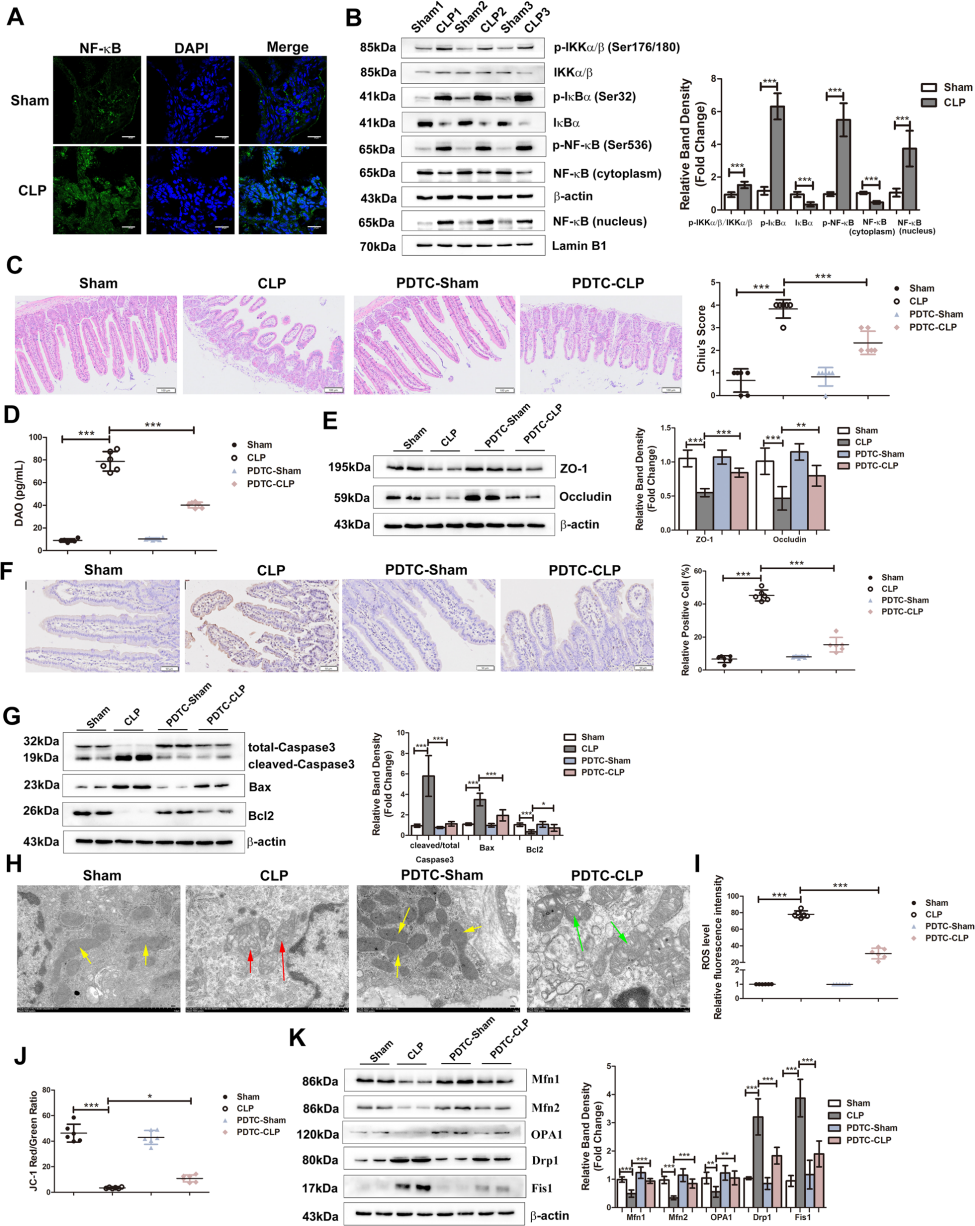
NF-κB signalling activation induces intestinal injury during sepsis. Mice were intraperitoneal injected with PDTC (100 mg/kg BW) and then subjected to CLP. **A** The location of NF-κB was detected after the indicated treatment. NF-κB is stained green; the nuclei are stained blue. Scale bar indicates 20 μm. **B** Representative western blots of indicated proteins in NF-κB signal shown on left and the right graph shows the relative band densities. **C** Microphotographs of H&E-stained intestine sections from the indicated groups. The scale bar represents 100 μm. The right graph shows the Chiu’s score of each group. **D** The serum concentration of DAO in each group. **E** The levels of ZO-1 and Occludin were analysed by Western blots. **F** Representative images of TUNEL staining. The graph shows the percentage of TUNEL-positive cells. The scale bar represents 50 μm. **G** The levels of apoptotic markers were measured by Western blots. The graph shows the relative band densities. **H** Morphological alterations in mitochondria were examined by TEM (scale bar = 1.0 μm). Yellow arrows indicate the relatively normal mitochondrial shape in the sham group. Red arrows indicate deformed mitochondria with the loss of clearly defined cristae. Green arrows indicate the rescued mitochondrial shape. **I** The relative fluorescence intensity of ROS in the intestine. **J** The ratio of JC-1 red/green fluorescence intensity which reflecting the MMP level was shown in graph. **K** Representative Western blots showing mitochondrial fusion (Mfn1, Mfn2, OPA1) and fission (Drp1 and Fis1) markers on the left, and the graph on the right shows the relative band densities. The data in the graphs are expressed as the mean ± SD. *P < 0.05, **P < 0.01, ***P < 0.001. (n = 6)

As a classic regulator of inflammation, NF-κB activity was measured during sepsis. We first investigated the localization of NF-κB (p65) in the intestinal epithelium, and the results showed that NF-κB (p65) was mainly located in the cytoplasm in sham mice, while most NF-κB (p65) translocated to the nucleus in CLP mice (Fig. 3A). Consistently, the protein level of NF-κB (p65) was increased in the nucleus and decreased in the cytoplasm in the CLP group (Fig. 3B). NF-κB activation involves the phosphorylation of IKKα/β, which phosphorylates IκBα, leading to the subsequent ubiquitination and degradation of IκBα and the translocation of NF-κB into the nucleus. The protein expression of p-IKKα/β, IKKα/β, p-IκBα, IκBα and p-P65 in the intestine was analysed by Western blotting. The results showed an upwards trend in p-IKK α/β,p-IκBα and p-p65 expression, while IκBα was decreased (Fig. 3B). These results showed activation of the IKK/IκB/NF-κB pathway in the intestine during sepsis. Subsequently, mice that underwent CLP were pre-treated with PDTC, an inhibitor of the NF-κB pathway, and the results showed that the integrity of the intestinal barrier was improved, as indicated by the restoration of the Chiu’s scores (Fig. 3C), serum DAO levels (Fig. 3D) and expression of tight junction proteins (ZO-1 and Occludin) (Fig. 3E). Moreover, PDTC pre-treatment reduced the number of apoptotic cells in intestinal epithelium (Fig. 3F), rescued the elevated expression of cleaved-Caspase3 and Bax, as well as the dropped level of Bcl2 (Fig. 3G). In addition, the impaired mitochondrial function presented as disrupted mitochondrial ultrastructure, increased ROS production, decreased MMP level, uplifted expression of mitochondrial fusion proteins (Mfn1, Mfn2, OPA1) and declined level of mitochondrial fission proteins (Drp1, Fis1) were all meliorative in CLP mice with PDTC pre-treatment (Fig. 3H–K).

### PLK1 negatively regulates the activity of NF-κB signalling in the intestine during sepsis

**Fig. 4:**
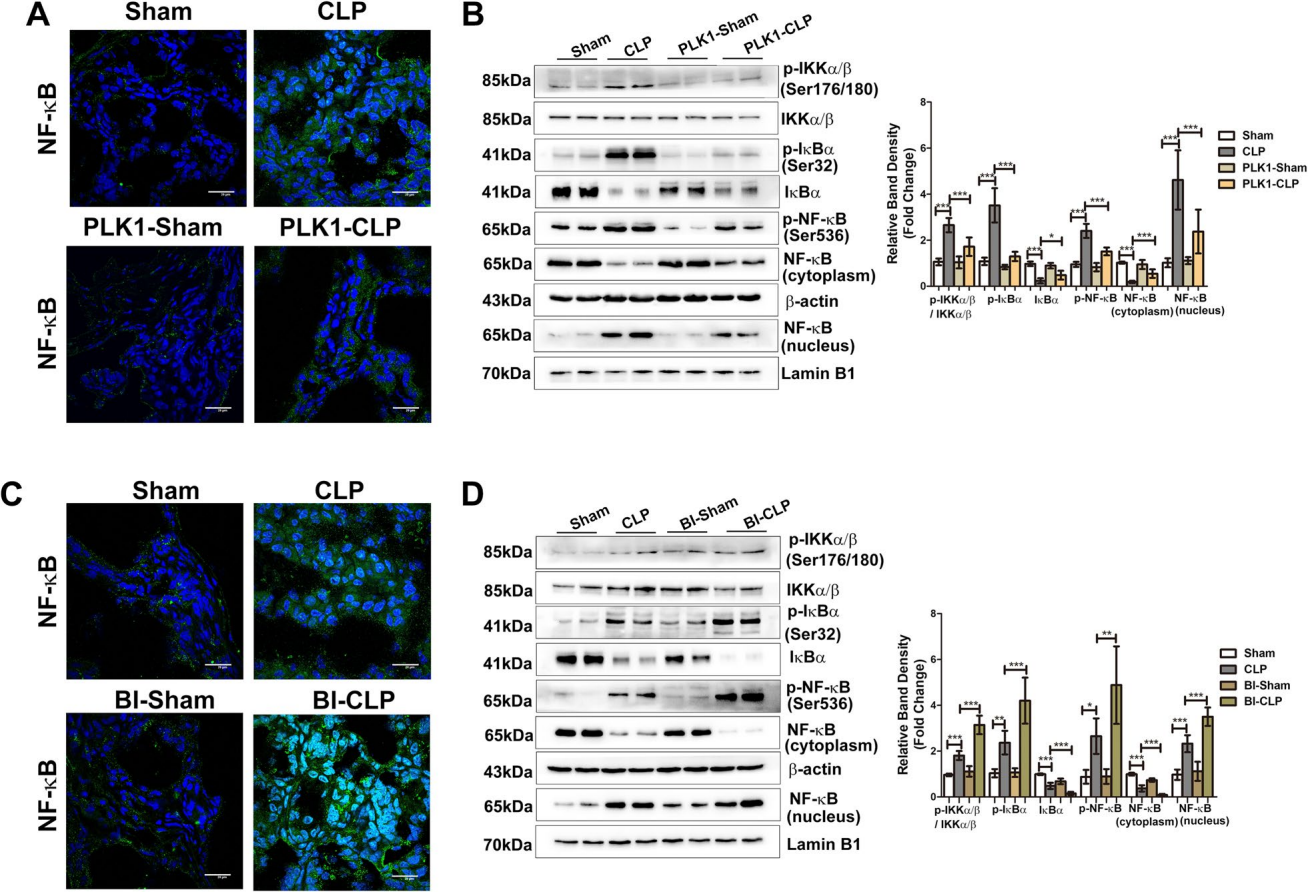
PLK1 negatively regulates the activity of NF-κB signalling in the intestine during sepsis. **A**, **C** The localization of NF-κB was examined after the indicated treatments. NF-κB is stained green; the nuclei are stained blue. The scale bar represents 20 μm. **B**, **D** Representative Western blots showing the indicated proteins in the NF-κB pathway are on the left, and the graph on the right shows the relative band densities. The data in the graphs are expressed as the mean ± SD. *P < 0.05, **P < 0.01, ***P < 0.001. (n = 6)

The relationship between PLK1 and NF-κB was also investigated in this study. In the CAG-PLK1 + CLP group, enterocyte nuclear expression of p65 was decreased compared with that in the WT + CLP group (Fig. 4A, B). Moreover, the protein expression of p-IKKα/β,p-IκBα and p-p65 decreased, while IκBα protein expression increased in the CAG-PLK1 + CLP group compared to the WT + CLP group (Fig. 4B), indicating that NF-κB pathway activity declined in the CAG-PLK1 + CLP group. Consistently, in the BI2536 + CLP group, the results were reversed. The expression of p65 in the nucleus and p-IKKα/β,p-IκBα and p-p65 in the total protein fraction increased, while the expression of p65 in the cytoplasm and IκBα in the total protein fraction decreased (Fig. 4C, D), indicating excessive activation of the NF-κB pathway. These results suggest that NF-κB pathway activity is negatively regulated by PLK1.

### PLK1 physically interacts with TANK and inhibits TANK expression in the intestinal epithelium

**Fig. 5:**
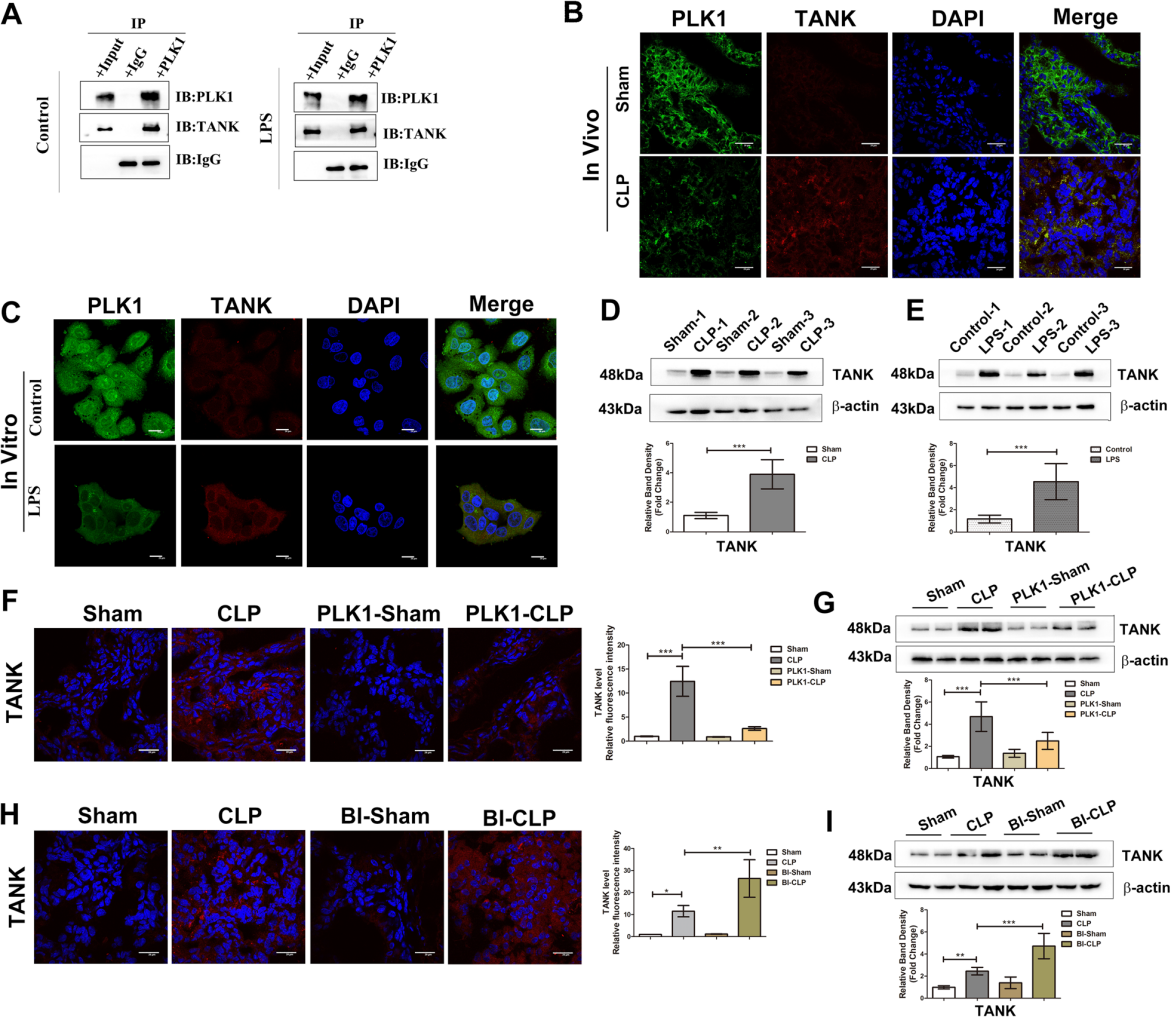
PLK1 physically interacts with TANK and inhibits TANK expression in the intestinal epithelium. **A** PLK1 physically interacted with TANK in Caco-2 cells with or without LPS treatment. **B**,** C** The location of PLK1 (green) and TANK (red) in the intestinal epithelium or Caco-2 cells after the indicated treatments. The nuclei are stained blue. The scale bar represents 20 μm. **D**,** E** Representative Western blots showing TANK after the indicated treatments in upper area, and the lower graph shows the relative band densities. **F**,** H** Representative microphotographs of TANK expression in the intestine after the indicated treatments (scale bar = 20 μm). The graph shows the relative fluorescence intensity. **G**, **I** Representative Western blots showing TANK after the indicated treatments in the upper area, and the lower graphs show the relative band densities. The data in the graphs are expressed as the mean ± SD. *P < 0.05, **P < 0.01, ***P < 0.001. (n = 6)

Previous studies revealed that TANK was an upstream regulator of NF-κB that interacts with PLK1 in HEK293 cells; thus, we hypothesized that PLK1 regulated NF-κB pathway activity via TANK in the intestinal epithelium. To test this hypothesis, we first performed Co-IP to examine the physical interaction of PLK1 with TANK in Caco-2 cells and found that endogenous PLK1 and TANK bound to each other in Caco-2 cells (Fig. 5A). Immunofluorescence analysis revealed the colocalization of PLK1 and TANK in the intestinal epithelium and Caco-2 cells (Fig. 5B, C). Furthermore, TANK expression was examined in the intestines of septic mice and LPS-treated Caco-2 cells, and the results showed increased expression of TANK in vivo and in vitro (Fig. 5D, E). However, the increase in TANK expression was inhibited in septic CAG-PLK1 mice (Fig. 5F, G) but was increased in BI2536-pretreated CLP mice (Fig. 5H, I), indicating that PLK1 inhibited the expression of TANK.

### Inhibiting TANK blocks NF-κB signalling and rescues LPS-induced mitochondrial dysfunction, apoptosis and high permeability in Caco-2 cells

**Fig. 6:**
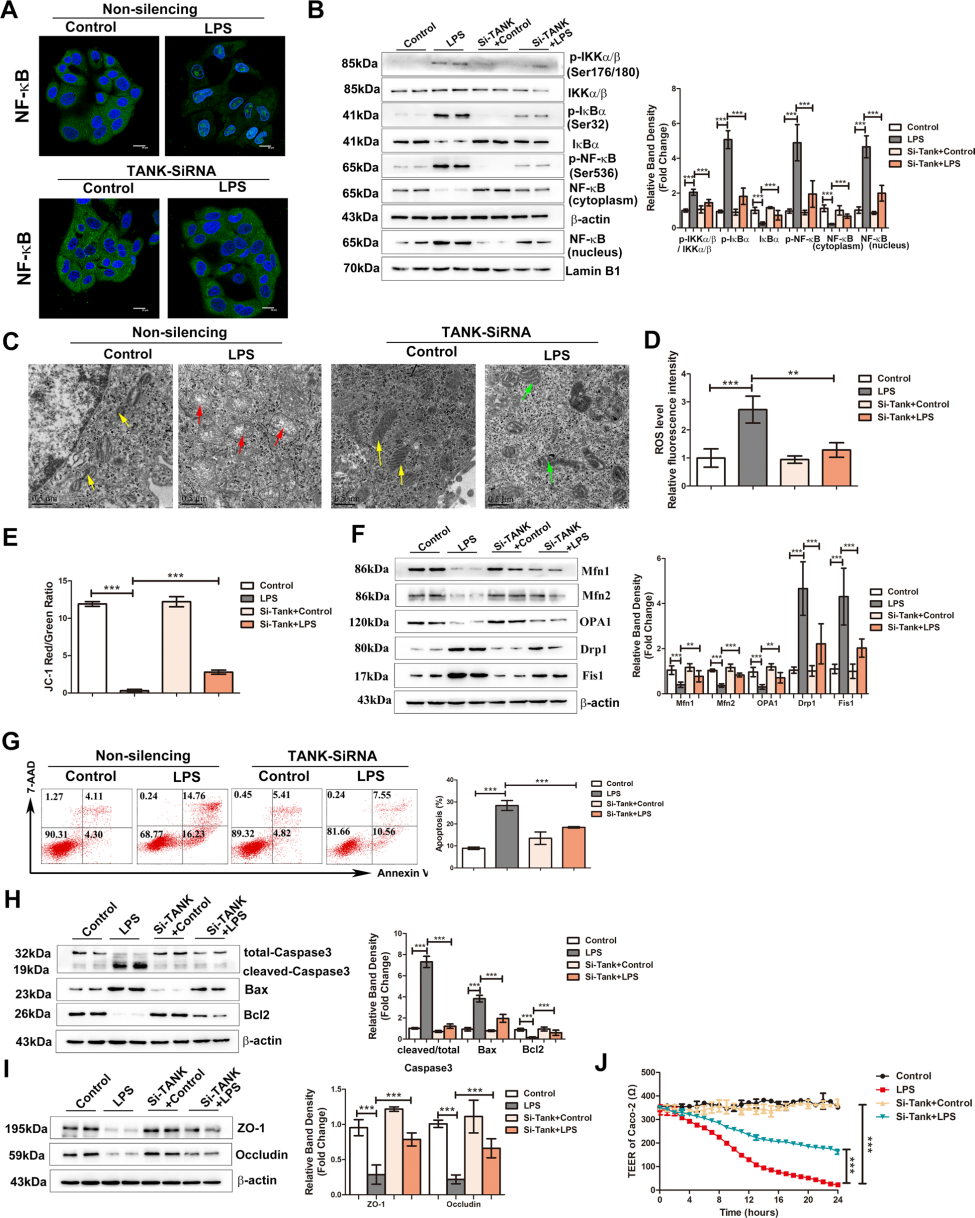
Inhibiting TANK blocks NF-κB signalling and rescues LPS-induced mitochondrial dysfunction, apoptosis and high permeability in Caco-2 cells. Caco-2 cells were transiently transfected with TANK-specific siRNA or control siRNA for 24 h and then treated with LPS (50 μg/mL) for 24 h. **A** The location of NF-κB was examined after the indicated treatments. NF-κB is stained green; the nuclei are stained blue. The scale bar represents 20 μm. **B** Representative Western blots showing the indicated proteins in the NF-κB pathway are on the left, and the graph on the right shows the relative band densities. **C** Morphological alterations in mitochondria were examined by TEM (scale bar = 0.5 μm). Yellow arrows indicate the relatively normal mitochondrial shape in the sham group. Red arrows denote deformed mitochondria with the loss of clearly defined cristae. Green arrows indicate the rescued mitochondrial shape. **D** The relative fluorescence intensity of ROS in the Caco-2 cells. **E** The ratio of JC-1 red/green fluorescence intensity which reflecting the MMP level was shown in graph. **F** Representative Western blots showing mitochondrial fusion (Mfn1, Mfn2, OPA1) and fission (Drp1 and Fis1) markers on the left, and the graph on the right shows the relative band densities. **G** Apoptosis was analysed by Annexin V-FITC/7-AAD double-labelling assays. The graph shows the proportion of apoptotic cells. **H** The levels of apoptotic markers were measured by Western blots. The graph shows the relative band densities. **I** The levels of ZO-1 and Occludin were analysed by Western blotting. The graph shows the relative band densities. **J** TEER levels in each group. The data in the graphs are expressed as the mean ± SD. Each result was replicated in three independent experiments. **P < 0.01, ***P < 0.001

To verify the effect of TANK on NF-κB activity, Caco-2 cells were transfected with Si-TANK and then incubated with LPS. The efficiency of Si-TANK silencing in Caco-2 cells was also measured (Additional file 1: Fig. S2A). The NF-κB pathway was markedly activated in LPS-treated Caco-2 cells, as evidenced by the increased nuclear translocation of p65, elevated expression of p-IKKα/β,p-IκBα and p-p65 and decreased expression of IκBα in the total protein fraction (Fig. 6A, B). However, the nuclear expression p65, p-IKKα/β,p-IκBα, and p-p65 in the cells decreased expression and IκBα expression increased in LPS-treated Caco-2 cells transfected with Si-TANK (Fig. 6A, B), indicating the suppression of NF-κB activity. Moreover, compared with unsilenced transfected cells, Si-TANK-transfected cells that were treated with LPS exhibited recovery of mitochondrial structure (Fig. 6C), reduced production of ROS (Fig. 6D), improved levels of MMP (Fig. 6E), increased expression of mitochondrial fusion proteins (Mfn1, Mfn2, OPA1) and decreased levels of mitochondrial fission proteins (Drp1, Fis1) (Fig. 6F), indicating the recovery of impaired mitochondrial function. Moreover, with Si-TANK transfection, the percentage of LPS-induced apoptotic cells declined (Fig. 6G), the levels of cleaved-Caspase3 and Bax decreased, and the expression of Bcl2 increased (Fig. 6H). The levels of tight junction proteins (ZO-1 and Occludin) and TEER were also improved in Si-TANK transfected Caco-2 cells (Fig. 6I, J). These results suggest that TANK knockdown inhibited NF-κB activity and ameliorated mitochondrial dysfunction, apoptosis and high permeability in LPS-treated Caco-2 cells.

### TANK overexpression impairs the protective effect of PLK1 on LPS-induced mitochondrial dysfunction, apoptosis and high permeability in Caco-2 cells

**Fig. 7:**
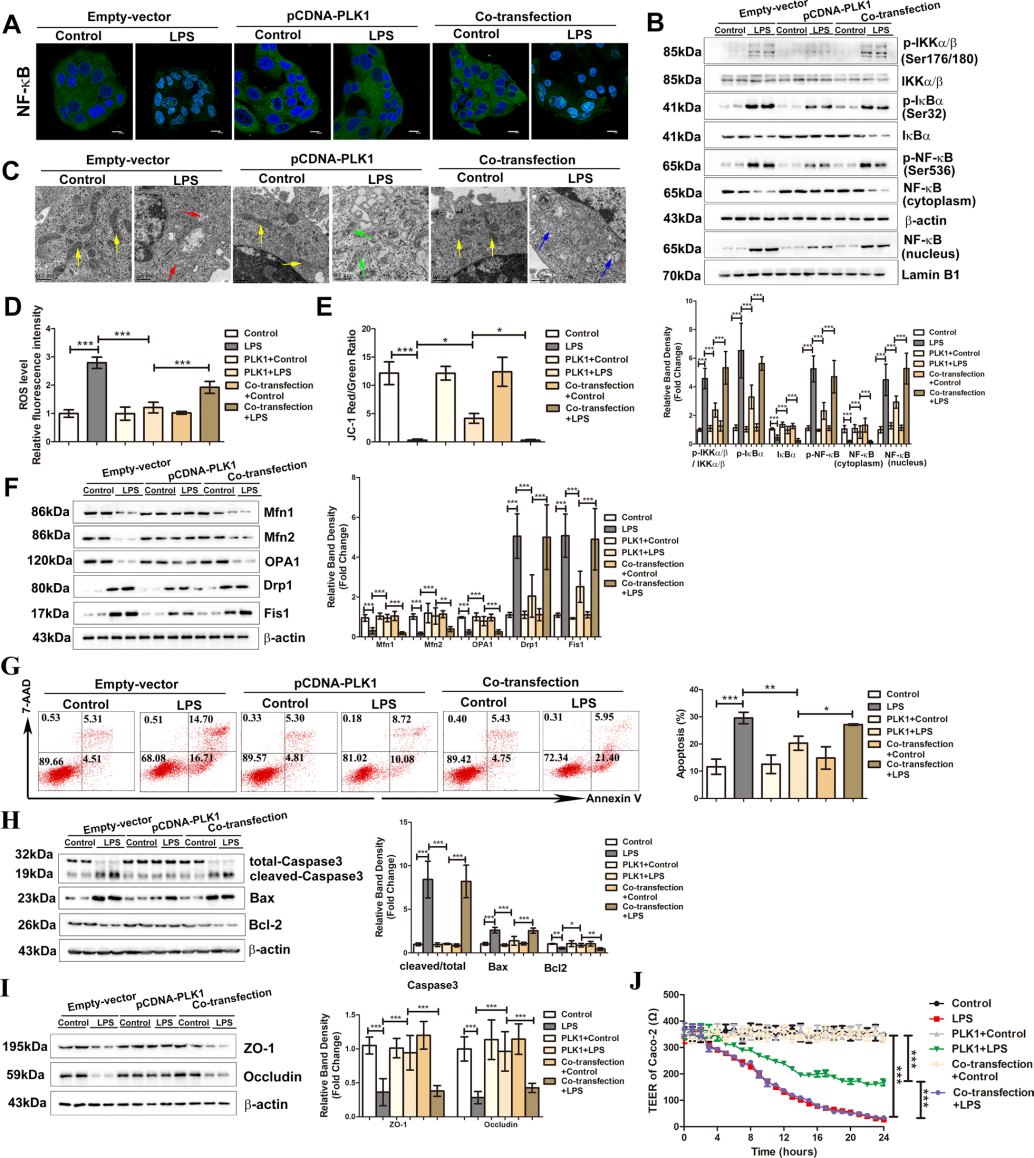
TANK overexpression impairs the protective effect of PLK1 on LPS-induced mitochondrial dysfunction, apoptosis and high permeability in Caco-2 cells. Caco-2 cells were transiently transfected with pCDNA3.1-PLK1 (a PLK1 knock-in plasmid) or/and pCDNA3.1-TANK (a TANK knock-in plasmid) for 24 h and then treated with LPS (50 μg/mL) for 24 h. **A** The location of NF-κB was examined after the indicated treatments. NF-κB is stained green; the nuclei are stained blue. The scale bar represents 20 μm. **B** Representative Western blots showing the indicated proteins in the NF-κB pathway, and the lower graph shows the relative band densities. **C** Morphological alterations in mitochondria were examined by TEM (scale bar = 0.5 μm). Yellow arrows indicate the relatively normal mitochondrial shape in the sham group. Red arrows denote deformed mitochondria with loss of clearly defined cristae. Green arrows indicate the rescued mitochondrial shape. Blue arrows indicate the worsened damage to mitochondrial shape. **D** The relative fluorescence intensity of ROS in the Caco-2 cells. **E** The ratio of JC-1 red/green fluorescence intensity which reflecting the MMP level was shown in graph. **F** Representative Western blots showing mitochondrial fusion (Mfn1, Mfn2, OPA1) and fission (Drp1 and Fis1) markers on the left, and the graph on the right shows the relative band densities. The data in the graphs are expressed as the mean ± SD. **G** Apoptosis was analysed by Annexin V-FITC/7-AAD double-labelling assays. The graph shows the proportion of apoptotic cells. **H** The levels of apoptotic markers were measured by Western blots. The graph shows the relative band densities. **I** The levels of ZO-1 and Occludin were analysed by Western blots. The graph shows the relative band densities. **J** TEER levels in each group. The data in the graphs are expressed as the mean ± SD. Each result was replicated in three independent experiments. *P < 0.05, **P < 0.01, ***P < 0.001

The protective effect of PLK1 on sepsis-induced intestinal barrier dysfunction was shown in CAG-PLK1 CLP mice. These findings were verified in vitro by transfecting Caco-2 cells with pCDNA3.1-PLK1 (a PLK1 knock-in plasmid) and incubating the cells with LPS. After pCDNA3.1-PLK1 transfection, the activity of NF-κB was suppressed, as shown by the reduced nuclear translocation of p65, decreased expression of p-IKKα/β,p-IκBα and p-p65 and elevated expression of IκBα in the total protein fraction (Fig. 7A, B). Moreover, mitochondrial structure, ROS production, MMP and the balance of mitochondrial dynamics were improved in PLK1-overexpressing Caco-2 cells (Fig. 7C–F). The percentage of apoptotic cells was reduced, the levels of cleaved-Caspase3 and Bax decreased, and the expression of Bcl2 increased (Fig. 7G, H). The permeability of cells decreased,which was associated with reduced TEER levels and increased expression of tight junction proteins (ZO-1 and Occludin) (Fig. 7I, J). Subsequently, Caco-2 cells were co-transfected with pCDNA3.1-PLK1 and pCDNA3.1-TANK (a TANK knock-in plasmid) and then treated with LPS. The transfection efficiency of pCDNA3.1-PLK1 and pCDNA3.1-TANK in Caco-2 cells were also measured (Additional file 1: Fig. S2B, C).Compared with cells that were transfected with pCDNA3.1-PLK1 alone, cells in the co-transfection group showed increased NF-κB signal activity (Fig. 7A, B), worsened mitochondrial impairments (Fig. 7C–F), increased apoptosis (Fig. 7G, H) and increased cell permeability (Fig. 7I, J), indicating that the protective effect of PLK1 was inhibited in co-transfected LPS-treated Caco-2 cells.

## Discussion

**Fig. 8:**
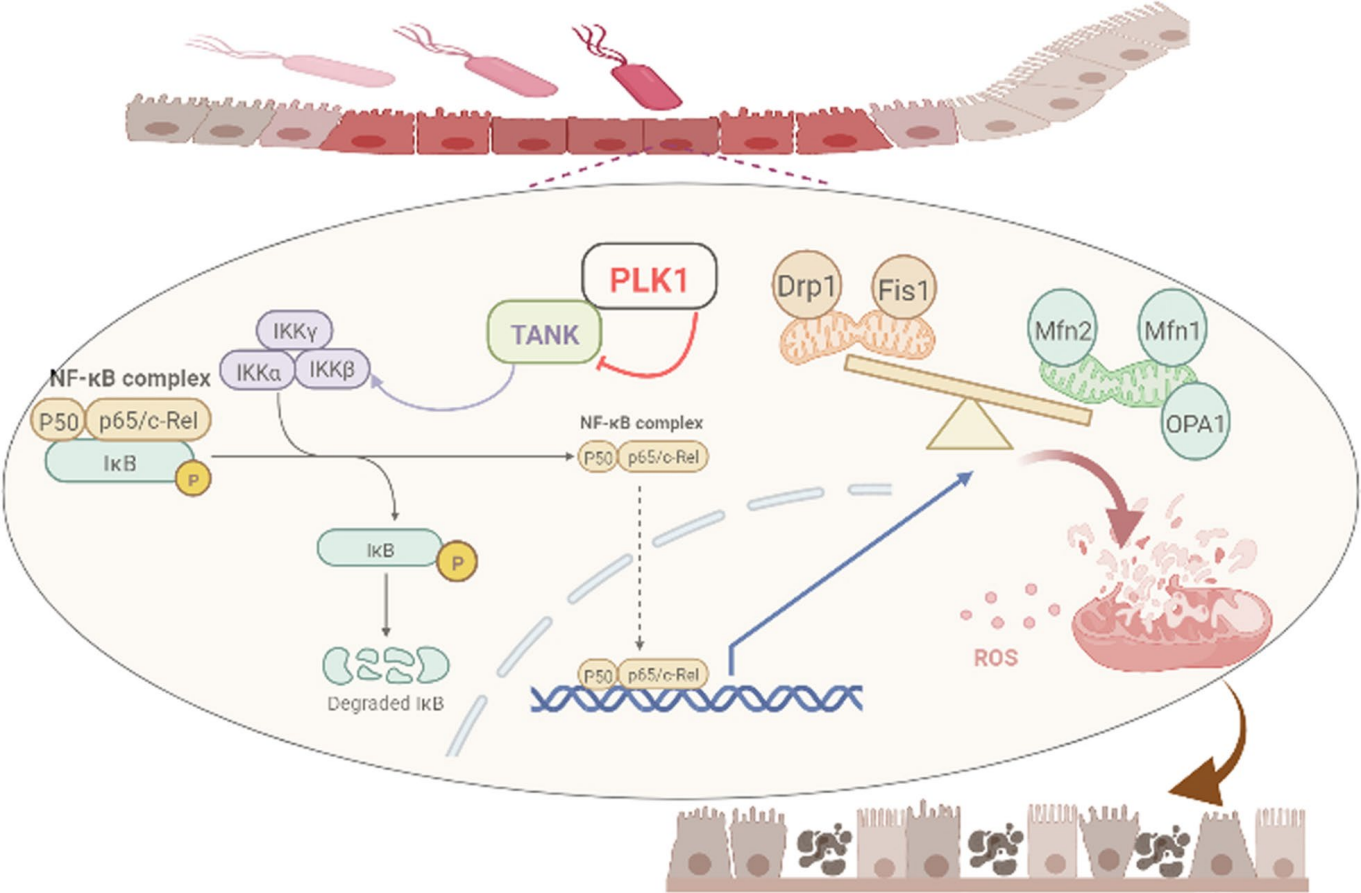
Schematic showing the regulatory mechanism of PLK1/TANK/NF-κB signalling in sepsis-induced intestinal barrier dysfunction. Sepsis/LPS downregulates PLK1 expression in the intestinal epithelium, weakening the inhibitory effect of PLK1 on TANK and increasing TANK expression, which subsequently activates NF-κB signalling. Activated NF-κB disrupts the balance in mitochondrial dynamics, destroys mitochondrial function through increased ROS production, induces apoptosis in the intestinal epithelium and ultimately leads to intestinal barrier damage.

In this study, we showed that PLK1 protects against sepsis-induced intestinal barrier dysfunction by improving the imbalance in mitochondrial fusion and fission and reducing apoptosis in the intestinal epithelium. Moreover, our findings demonstrated that the protective effect of PLK1 relies on its negative regulation of NF-κB signalling. Further examination revealed that PLK1 interacts with TANK, an upstream regulator of NF-κB, and inhibits the expression of TANK to regulate NF-κB signalling (Fig. 8). Thus, the PLK1/TANK/NF-κB axis was shown to play a crucial role in sepsis-induced intestinal barrier dysfunction and might be a potential therapeutic target in the clinic.

Mitochondria are crucial organelles in sepsis because their dysfunction can lead to loss of function in different organs, such as the lung, kidney and heart (Shi et al. 2019; Li et al. 2021; van der Slikke et al. 2021). The present study revealed the sepsis-induced mitochondrial damage was characterized by increased ROS, decreased MMP and disruption of mitochondrial dynamic balance in the intestinal epithelium. Mitochondrial dynamic balance depends on fission and fusion, which are mediated by conserved dynamin-related GTPase proteins including the fusion proteins optic atrophy 1 (OPA1), mitofusin1 (Mfn1) and mitofusin2 (Mfn2), as well as the fission protein dynamin-related protein 1 (Drp1) and its receptor mitochondrial fission protein 1 (Fis1) (Hoppins et al. 2007; Whitley et al. 2019). Our results showed that the expression of Drp1 and Fis1 was increased, while OPA1, Mfn1 and Mfn2 were decreased in the intestinal epithelium during sepsis, indicating increased mitochondrial fission and insufficient mitochondrial fusion.

Excessive fission and deficient fusion in mitochondria during sepsis result in the accumulation of mitochondrial fragmentation, which induces the production of ROS and permeabilizes the outer membrane, damaging mitochondrial function (Chan 2012).Furthermore, impaired mitochondria release cytochrome c, an essential component of the respiratory chain, into the cytosol to trigger apoptosis (Borutaite 2010; Kalpage et al. 2019). The B-cell lymphoma 2 (Bcl2) family mediates cytochrome c release, and cytochrome c in the cytosol can bind to apoptotic protease factor 1 (Apaf1), forming the apoptosome complex, activating caspase 3 and caspase 9, and resulting in apoptotic features such as DNA fragmentation and chromatin condensation (Shakeri et al. 2017). This study also verified excessive apoptosis in the intestinal epithelium, which was characterized by elevated expression of Bax and cleaved caspase 3, decreased Bcl2 expression and an increase in TUNEL-positive cells, which was accompanied by mitochondrial dysfunction during sepsis. Because an efficient intestinal barrier relies on structural integrity that is maintained by properly regulated rates of epithelial proliferation and death, dysregulated apoptosis in the intestinal epithelium can damage the tight junctions of cells, increase the permeability of the intestine and result in intestinal barrier dysfunction.

NF-κB signalling is known to play a pivotal role in inflammation by mediating the expression of inflammatory cytokines and chemokines (Lawrence 2009). The NF-κB family is composed of five members known as RelA/p65, RelB, c-Rel, NF-κB1 (p105/p50) and NF-κB2 (p100/p52), among which p50/p65 is the most representative dimer (Napetschnig et al. 2013). One sign of NF-κB signalling activation is the translocation of dimers from the cytoplasm into the nucleus. In the canonical pathway, NF-κB dimers are located in the cytoplasm bound to IκBα, an inhibitory protein in the IκB family. In response to stimulation, IκBα is phosphorylated by the IκB kinase (IKK) complex and degraded, and NF-κB dimers are released and translocate to the nucleus (Oeckinghaus et al. 2009). NF-κB signalling controls mitochondrial dynamics in various diseases. In insulin-treated cardiomyocytes, NF-κB regulates OPA1 expression and mitochondrial fusion (Parra et al. 2014). In fuel-oversupplied muscle cells, activation of the IKK–NF-κB axis promotes the expression of Drp1 and suppresses MFN2 levels, thus inducing mitochondrial fragmentation (Nisr et al. 2019). Incremental mitochondrial fragmentation can in turn increase NF-κB activity by phosphorylating IKK and IκBα through ROS accumulation (Huang et al. 2016). Similarly, the present results demonstrated that pharmacological inhibition of the NF-κB pathway not only ameliorated the changes in fission proteins (Drp1 and Fis1) and fusion proteins (OPA1, Mfn1 and Mfn2) but also improved mitochondrial function by rescuing MMP and ameliorating ROS production in the intestinal epithelium during sepsis.

TANK was identified as a TRAF-binding protein that possesses opposite regulatory properties in innate immune activation (Rothe et al. 1996). Its effect on the NF-κB pathway is also controversial because TANK can activate NF-κB signalling in cells expressing TRAF2, while TANK inhibits NF-κB activation by interacting with TRAFs (Cheng et al. 1996; Maruyama et al. 2012). Because the expression of TANK differs with different stimuli (Wang et al. 2015; Feng et al. 2018), we examined the level of TANK in the intestinal epithelium and verified that TANK expression was markedly increased in vitro and in vivo during sepsis. Furthermore, the relationship between TANK and the NF-κB pathway was examined in the intestinal epithelium by transfecting Caco-2 cells with Si-TANK. The results showed that TANK knockdown inhibited the activity of the IKK–NF-κB axis and ameliorated LPS-induced apoptosis and mitochondrial dysfunction, suggesting that TANK acts as a positive regulator of NF-κB signalling in the intestinal epithelium during sepsis.

PLK1 was shown to partly rescue LPS-induced apoptosis in vitro (Cao et al. 2018, 2021). In this study, inducible PLK1 knock-in mice were subjected to CLP, and the results showed the amelioration of mitochondrial dysfunction and apoptosis in the intestinal epithelium and improvements in intestinal permeability compared with the controls. Considering that the serine/threonine protein kinase PLK1 plays a critical role in mitosis and that mitochondria supply fuel during the cell cycle (Salazar-Roa et al. 2017), it is not surprising that a specific link exists between PLK1 and mitochondrial function. Mitochondrial structure is regulated by cyclin-dependent kinase (CDK) and the anaphase-promoting complex/cyclosome (APC/C), which promote mitotic entry and sister chromatid separation during cytokinesis (Mishra et al. 2014). Overexpression of PLK1, an upstream regulator of the cyclin B1/Cdk1 complex, could restore the cell cycle, which was disrupted by Fis1 depletion (Lee et al. 2014). The mitotic kinase Aurora A has also been reported to regulate Drp1 function by phosphorylating RalA, inducing mitochondrial fission and leading to improper segregation of mitochondria (Kashatus et al. 2011; Park et al. 2012). Our results revealed that inhibiting PLK1 with BI2536, an ATP binding domain inhibitor of PLK1, could damage mitochondrial dynamics in CLP mice, increasing the expression of fission proteins (Drp1 and Fis1) and decreasing the expression of fusion proteins (OPA1, Mfn1 and Mfn2).

Previous studies revealed a paradoxical relationship between PLK1 and NF-κB signalling in different types of cells (Higashimoto et al. 2008; Lin et al. 2011; Wu et al. 2019). The present findings showed that PLK1 acts as a negative regulator of NF-κB signalling by inhibiting the phosphorylation of IKKα/β and IκBα, thus inhibiting P65 translocation to the nucleus. Further examination identified the physical interaction and colocalization of PLK1 and TANK in the intestinal epithelium, which provides structural evidence that PLK1 controls the activity of NF-κB signalling via TANK. Research on the relationship between PLK1 and TANK is limited. Zhang reported that PLK1 was recruited to the IKK complex through TANK in HEK293 cells (Higashimoto et al. 2008; Lin et al. 2011; Wu et al. 2019). In this study, we found that the expression of TANK was suppressed in septic PLK1 knock-in mice. TANK overexpression abolished the protective effect of PLK1 on LPS-induced mitochondrial dysfunction, apoptosis and hyperpermeability in the intestinal epithelium by activating NF-κB signalling. Therefore, we hypothesize that PLK1 negatively regulates NF-κB signalling by binding and inhibiting TANK in the intestinal epithelium during sepsis.

In conclusion, our work demonstrates that PLK1 protects against sepsis-induced intestinal barrier dysfunction via TANK/NF-κB signalling by rescuing mitochondrial dysfunction and inhibiting apoptosis in the intestinal epithelium. The PLK1/TANK/NF-κB pathway might serve as a novel therapeutic target for the treatment or prevention of intestinal dysfunction. Further study should be paid attention on the application of PLK1 as the diagnostic or prognostic indicator of intestinal dysfunction in clinic.

## Supplementary Information

**Additional file 1.** The effiencey of PLK1 inhibition on sepsis-induced intestinal injury and the details of primary antibodies for Western blots.

## Data Availability

All data needed to evaluate the conclusions in the paper are present in the paper and/or the Additional Materials. Additional data related to this paper may be requested from the authors.

## Abbreviations

PLK1: Polo-like kinase 1
NF-κB: Nuclear factor-κB
TANK: TRAF family member-associated NF-κB activator
TRAF: TNF receptor associated factor
CLP: Caecal ligation and puncture
LPS: Lipopolysaccharide
PDTC: Pyrrolidinedithiocarbamate ammonium
WT: Wild-type
SPF: Specific pathogen-free
ELISA: Enzyme-linked immunosorbent assay
HE staining: Haematoxylin and eosin staining
TUNEL: Terminal deoxynucleotidyl transferase (TdT)-mediated dUDP-biotin nick end labelling
FBS: Foetal bovine serum
ROS: Reactive oxygen species
MMP: Mitochondrial membrane potential
TEER: Transepithelial electrical resistance
TEM: Transmission electron microscopy
DAO: Diamine oxidase
IKK: IκB kinase
Bcl2: B-cell lymphoma 2
Bax: BCL2-Associated X
CDK: Cyclin-dependent kinase
APC/C: Anaphase-promoting complex/cyclosome
