# Supplementary material for: PLK1 protects intestinal barrier function during sepsis by targeting mitochondrial dynamics through TANK-NF-κB signalling
Source: Mol Med. 2022 Dec 29;28:163. doi: 10.1186/s10020-022-00597-z (PMC9801534; doi:10.1186/s10020-022-00597-z)
Supplement: Supplementary file 1 — Additional file 1. The effiencey of PLK1 inhibition on sepsis-induced intestinal injury and the details of primary antibodies for Western blots. [file 10020_2022_597_MOESM1_ESM.docx]

Supplementary Materials for

**PLK1 protects intestinal barrier function during sepsis**

**by targeting mitochondrial dynamics through**

**TANK-NF-κB signalling**

Ying-Ya Cao^1,2#^, Yuan Zhang^1#^, Wuyun Gerile^1#^, Yan Guo^1^, Li-Na Wu^1^, Li-Li Wu^1^, Kai Song^1^, Wei-Hua Lu^2^, Jian-Bo Yu^1^*

1. Department of Anesthesiology and Critical Care Medicine, Tianjin Nankai Hospital, Tianjin Medical University, Tianjin, 300100, China. 2. Department of Critical Care Medicine, The First Affiliated Hospital of Wannan Medical College, Wuhu 241001, Anhui, China.

E-mail addresses of the co-authors:

Ying-Ya Cao E-mail: caoyingya1990@126.com

Yuan Zhang E-mail: [13642066361@126.com](mailto:13642066361@126.com)

Wuyun Gerile E-mail: [58847727@qq.com](mailto:58847727@qq.com)

Yan Guo E-mail: guoyanfuwai2015@163.com

Li-Na Wu E-mail: wln6527@126.com

Li-Li Wu E-mail: 1710368375@qq.com

Kai Song E-mail: 371938167@qq.com

Wei-Hua Lu E-mail:[lwh683@126.com](mailto:lwh683@126.com)

Jian-Bo Yu* E-mail: [30717008@nankai.edu.cn](mailto:30717008@nankai.edu.cn)

# These authors contributed equally to this work

**Corresponding author**: Jian-Bo Yu (E-mail: [30717008@nankai.edu.cn](mailto:30717008@nankai.edu.cn))


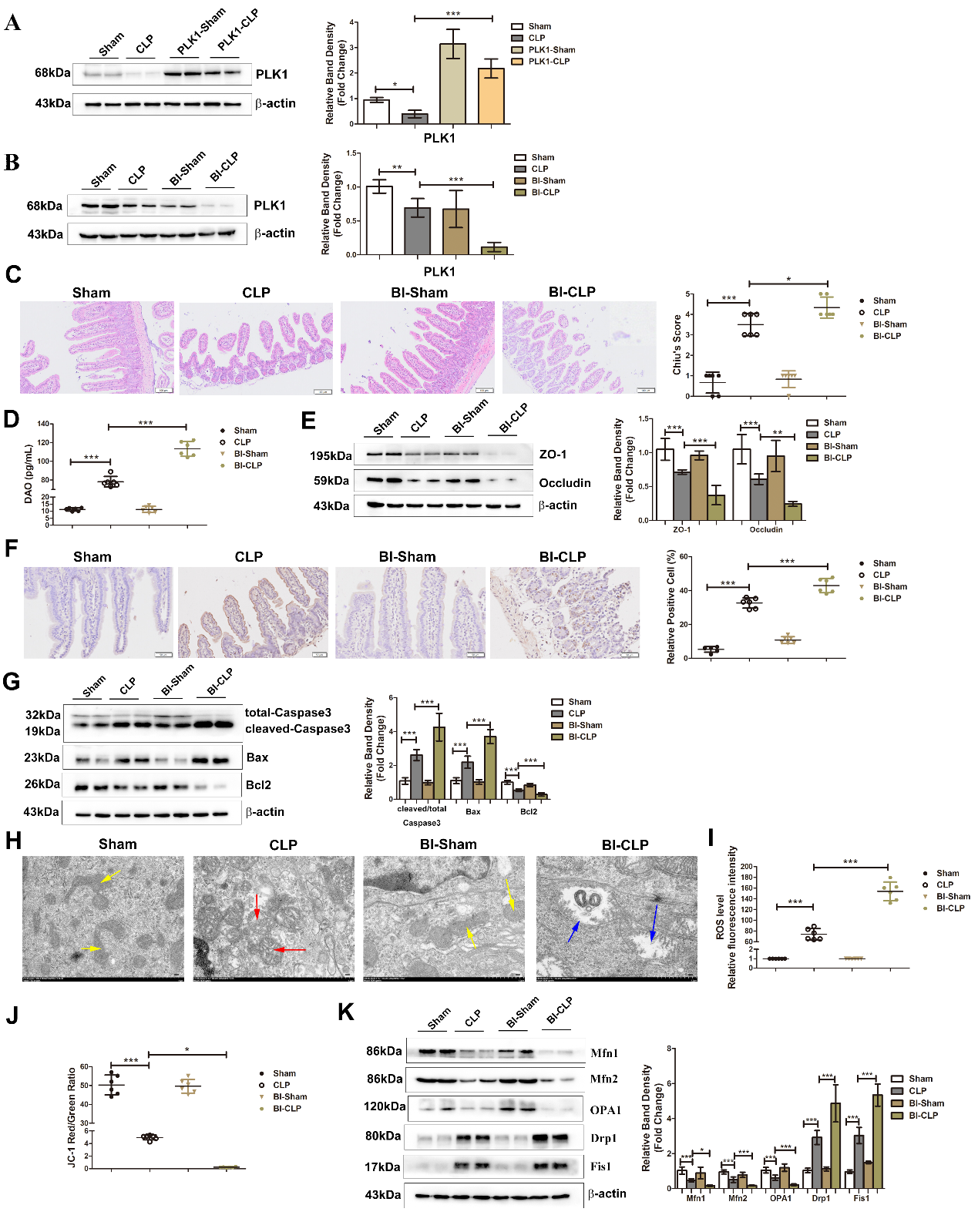


**Supplementary Figure 1 (Fig. S1) Inhibition of PLK1 deteriorates sepsis-induced intestinal injury**. Mice were intraperitoneal injected with BI2536 (10 mg/kg BW) 24 h before CLP operation. **A, B** The expression level of PLK1 were measured after the above treatments. The graph shows the relative band densities. **C** Microphotographs of H&E-stained intestine sections from the indicated groups. The scale bar represents 100 μm. The Chiu’s score of each group shown right. **D** Serum concentrations of DAO in each group. **E** The levels of ZO-1 and Occludin were analysed by Western blots. The graph shows the relative band densities. **F** Representative images of TUNEL staining after the indicated treatments. The graph shows the percentage of TUNEL-positive cells. The scale bar represents 50 μm. **G** The levels of apoptotic markers were measured by Western blots. The graph shows the relative band densities. **H** Morphological alterations in mitochondria were examined by TEM (scale bar = 1.0 μm). Yellow arrows indicate the relatively normal mitochondrial shape in the sham group. Red arrows denote deformed mitochondria with the loss of clearly defined cristae. Green arrows indicate rescued mitochondrial shape. **I** The relative fluorescence intensity of ROS in the intestine. **J** The ratio of JC-1 red/green fluorescence intensity which reflecting the MMP level was shown in graph. **K** Representative Western blots showing mitochondrial fusion (Mfn1, Mfn2, OPA1) and fission (Drp1 and Fis1) on the left, and the right graph show the relative band densities. The data in the graphs are expressed as the mean ± SD. **P* < 0.05, ***P* < 0.01, ****P* < 0.001. (n = 6).

**
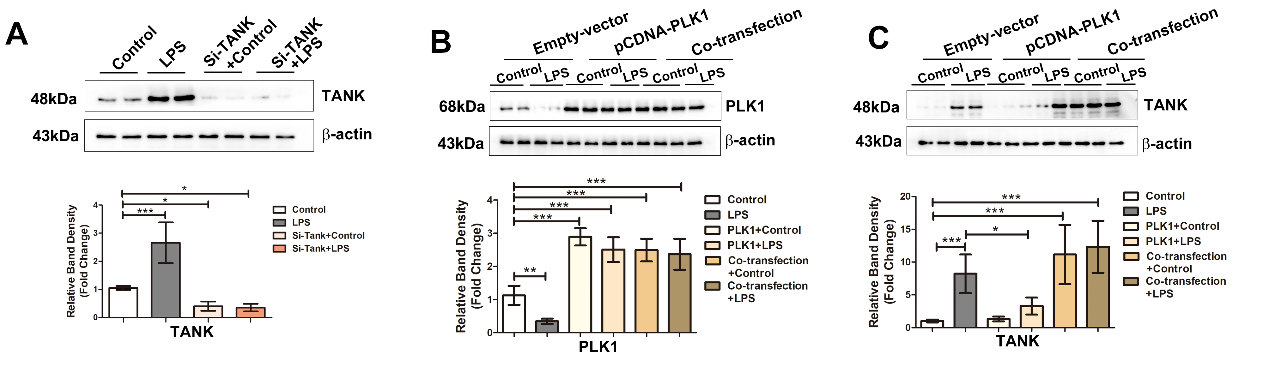
**

**Supplementary Figure 2 (Fig. S2) The transfection efficiency of Si-RNA or plasmid in Caco-2 cells.** Caco-2 cells were transfected with indicated Si-RNA or plasmid and the corresponding proteins expression were detected. (A) The expression level of TANK with indicated treatments. (B) The expression level of PLK1 with indicated treatments.(C) The expression level of TANK with indicated treatments. The graph shows the relative band densities. Each result was replicated in three independent experiments. **P* < 0.05, ***P* < 0.01, ****P* < 0.001.

**Supplementary Table 1: The details of primary antibodies for Western blots**

| **Antibody** | **Manufacturer** | **Catalogue numbers** | **Dilution** |
| --- | --- | --- | --- |
| anti-PLK1 | Abcam | [ab17057](https://www.abcam.cn/plk1-antibody-36-298-ab17057.html) | 1:1000 |
| anti-TANK | Santa Cruz | sc-166643 | 1:1000 |
| anti-p-IKKα/β(Ser176/180) | Cell Signaling Technology | 2697 | 1:500 |
| anti-IKKα/β | Abcam | [ab178870](https://www.abcam.cn/plk1-antibody-36-298-ab17057.html) | 1:1000 |
| anti-p-IκBα(Ser32) | Santa Cruz | sc-8404 | 1:1000 |
| anti-IκBα | Santa Cruz | sc-1643 | 1:1000 |
| anti- p-NF-κB(Ser536) | Santa Cruz | sc-136548 | 1:1000 |
| anti-NF-κB | Cell Signaling Technology | 8242 | 1:500 |
| anti-Caspase3 | Proteintech | 66470-2-Ig | 1:1000 |
| anti-Bcl2 | Santa Cruz | sc-7382 | 1:1000 |
| anti-Bax | Santa Cruz | sc-7480 | 1:1000 |
| anti-ZO-1 | Proteintech | 21773-1-AP | 1:1000 |
| anti-Occludin | Proteintech | 13409-1-AP | 1:1000 |
| anti-Mfn1 | Santa Cruz | sc-166644 | 1:1000 |
| anti-Mfn2 | Santa Cruz | sc-515647 | 1:1000 |
| anti-OPA1 | Santa Cruz | sc-393296 | 1:1000 |
| anti-Drp1 | Santa Cruz | sc-271583 | 1:1000 |
| anti-Fis1 | Santa Cruz | sc-376447 | 1:1000 |
| anti-Lamin B1 | Abcam | ab16048 | 1:5000 |
| anti-β-actin | Proteintech | 20536-1-AP | 1:5000 |
